# Polymorphism at codon 31 of CDKN1A (p21) as a predictive factor for bevacizumab therapy in glioblastoma multiforme

**DOI:** 10.1186/s12885-023-11400-5

**Published:** 2023-09-20

**Authors:** Wen-Yu Cheng, Chiung-Chyi Shen, Yea-Jiuen Liang, Ming-Tsang Chiao, Yi-Chin Yang, Wan-Yu Hsieh, Cheng-Hui Lin, Jun-Peng Chen

**Affiliations:** 1 Department of Minimally Invasive Skull Base Neurosurgery, Neurological Institute, Taichung Veterans General Hospital, Taichung city, Taiwan; 2 Department of Physical Therapy, Hung Kuang University, Taichung city, Taiwan; 3 Institute of Biomedical Sciences, National Chung Hsing University, Taichung city, Taiwan; 4 Department of Post-Baccalaureate Medicine, College of Medicine, National Chung Hsing University, Taichung city, Taiwan; 5 Basic Medical Education, Central Taiwan University of Science and Technology, Taichung city, Taiwan; 6 Biostatistics Task Force, Taichung Veterans General Hospital, Taichung city, Taiwan

**Keywords:** Glioblastoma, CDKN1A c.93C > A, Bevacizumab, Polymorphism, PCR–RFLP

## Abstract

Glioblastoma (GBM), a prevalent and malignant brain tumor, poses a challenge in surgical resection due to its invasive nature within the brain parenchyma. CDKN1A (p21, Waf-1), a cyclin-dependent kinase inhibitor, plays a pivotal role in regulating cell growth arrest, terminal differentiation, and apoptosis. The existence of natural variants of CDKN1A has been associated with specific cancer types. In this retrospective study, our objective was to identify polymorphic variants of CDKN1A, specifically c.93C > A (codon 31 Ser31Arg), and investigate its potential impact within the scope of bevacizumab therapy for glioblastoma multiforme. This study involved a cohort of 139 unrelated adult Chinese GBM patients in Taiwan. Genomic DNA extracted from tumor samples was utilized for genotyping using the polymerase chain reaction (PCR) restriction fragment length polymorphism method (PCR–RFLP analysis). Through unconditional logistic regression analysis, odds ratios (ORs) with corresponding 95% confidence intervals (CIs) were calculated. Our findings unveiled that among these GBM patients, the distribution of codon 31 polymorphisms was as follows: 23.02% were Serine homozygotes (Ser/Ser), 27.34% were Arginine homozygotes (Arg/Arg), and 49.64% were Serine/Arginine heterozygotes (Ser/Arg). While CDKN1A c.93C > A polymorphisms did not exhibit a direct association with overall survival in GBM patients, noteworthy survival benefits emerged among individuals with Arg/Arg and Arg/Ser genotypes who received combined concurrent chemoradiotherapy (CCRT) and bevacizumab treatment compared to those who underwent CCRT alone. Our findings indicate a significant involvement of the CDKN1A c.93C > A polymorphism in the development and onset of GBM, offering potential implications for the early prognostication of bevacizumab therapy outcomes.

**Supplementary Information:**

The online version contains supplementary material available at 10.1186/s12885-023-11400-5.

## Introduction

Glioblastoma multiforme (GBM) is an aggressive brain tumor known for its high resistance to treatment. Despite multiple attempts using various immunotherapeutic approaches and combinations [1], GBM remains incurable. Patients with glioblastoma have a poor prognosis, with a median survival of 14.6 months and a 2-year survival rate of less than 26.5% [2]. In a recent study conducted on the Taiwanese population, it was found that the 1-year survival rate of GBM was only 50.3%, which was significantly lower (24.0%) compared to the 2-years survival rate [3]. There are two major contributing factors to this outcome. Firstly, GBM frequently recurs and metastasizes due to the rapid proliferation of infiltrative residual tumor cells. Secondly, tumor cells that are resistant to current chemotherapy contribute to tumor regrowth and recurrence, which is often inevitable. Therefore, there is an urgent need to explore approaches that can improve the outcomes of GBM patients.

In 2009, the Food and Drug Administration (FDA) granted accelerated approval for bevacizumab (BEV), also known as Avastin, a humanized anti-VEGF monoclonal immunoglobulin 1 (IgG1) antibody. BEV inhibits angiogenesis by disrupting the VEGF/VEGF-receptor signaling pathway, thereby exerting indirect antitumor activity [4]. Currently, BEV is used for the treatment of recurrent glioblastoma multiforme (rGBM) [5]. Subsequent clinical studies also have demonstrated the effectiveness of bevacizumab in increasing the objective response rate and median progression-free survival in patients with rGBM [6]. However, its contribution to extending patient survival in newly diagnosed GBM or progressive GBM has not been established in several clinical trials [7–9]. Nonetheless, prolonged progression-free survival (PFS) and overall survival (OS) have been reported in the recurrence setting with bevacizumab alone or in combination with other chemotherapy [10]. These results highlight the controversial findings regarding the impact of bevacizumab on PFS and OS.

Due to the complex pathogenesis and multiple genetic heterogeneities, tumor suppressor gene p53, and its downstream effecter p21 are believed to play significant roles in cancer development. The p21^waf1/cip1^ gene (CDKN1A, Cyclin-Dependent Kinase Inhibitor 1A; OMIM:116,899; hereafter referred to as CDKN1A) encodes an essential cell cycle regulatory protein that inhibits cell cycle progression from G1 to S phase, thereby regulating cell proliferation, growth arrest, and apoptosis. As the primary downstream regulator of the tumor suppressor p53, CDKN1A serves as a crucial link between p53 to cell-cycle arrest and DNA repair [11, 12]. Consequently, it has been suggested that CDKN1A may exert an influence on tumor genesis [13]. While CDKN1A gene mutations are rare in carcinoma [14], a decrease in CDKN1A expression is often associated with a poor prognosis [15–17]. This suggests that genetic polymorphisms in CDKN1A are likely to modulate its expression, thereby influencing the pathogenesis and initiation of carcinoma.

Single nucleotide polymorphisms (SNPs) in the human genome have been found to influence susceptibility to various types of cancer. Several studies have indicated that CDKN1A polymorphisms can impact protein expression and activity, and play a role in cancer susceptibility [14, 17]. The two most extensively studied CDKN1A polymorphisms are situated at codon 31, specifically CDKN1A c.93C > A (p.Ser31Arg), previously referenced as CDKN1A C98A, NM_000389.5 (CDKN1A):c.93C > A, and documented as dbSNP rs1801270 C > A. This polymorphism involves a transversion substitution, where the base changes from C to A, leading to a non-synonymous serine-to-arginine substitution in the CDKN1A protein. This alteration results in the loss of the Blp I restriction site and impacts the DNA-binding zinc finger motif. Another noteworthy polymorphism is detected in the CDKN1A 3'UTR c.*70C > T, previously known as CDKN1A 3’UTR (CDKN1A C70T, dbSNP rs1059234 C > T), involving a transition substitution that modifies the nucleotide from C to T. These polymorphisms, whether in isolation or in combination, are believed to exert influence on carcinogenesis [18–20]. Typically, these polymorphic changes lead to a reduction in the transcriptional activity of CDKN1A [21], consequently heightening susceptibility to cancer [22].

The impact of the Ser31Arg polymorphism on cancer risk has been extensively investigated in numerous molecular epidemiological studies. However, these studies have reported conflicting results, highlighting the need to further explore the underlying heterogeneity. To address this gap, we conducted a retrospective study focusing on the potential role of CDKN1A functional polymorphism as a predictive marker in patients with glioblastoma multiforme within the Taiwanese population. Through genotyping analysis of CDKN1A, we made a novel finding, establishing a significant association between GBM patient survival and the presence of either arginine homozygotes (Arg/Arg) or serine/arginine heterozygotes (Ser/Arg) at codon 31 of CDKN1A, particularly following bevacizumab therapy in a Chinese population.

## Materials and methods

### Subjects

The study protocols were approved by the Medical Ethics Committee of Taichung Veterans General Hospital (Approval number: CF17263B-4). This retrospective study included 139 GBM patients ranging in age from 20 to 92 years. GBM diagnosis was confirmed through pathological examinations. Samples were obtained from the Department of Minimally Invasive Skull Base Neurosurgery, Neurological Institute, Taichung Veterans General Hospital, from 2010 to 2022, comprising the primary study cohort. Informed consent was obtained from all subjects or their legal guardians prior to surgery, and the collected samples were promptly frozen. The informed consent process involved providing detailed information about the study's objectives, procedures, potential risks, and benefits to the participants. They were given ample opportunity to ask questions and clarify any concerns before voluntarily providing their consent to participate. The study protocol and informed consent procedure were reviewed and approved by the relevant institutional ethics committee to ensure compliance with ethical guidelines and standards. All GBM patients underwent surgical resection and received concurrent chemoradiotherapy with temozolomide (Temozolomide (TMZ): 75 mg/m2/d) (CCRT), followed by adjuvant TMZ (150–200 mg/m2/d). Bevacizumab (10 mg/kg intravenously every 2 weeks until disease progression) was administered only to patients with recurrent GBM. The validation cohort consisted of 139 cases selected from the primary cohort based on the following criteria: (1) availability of follow-up data and samples, and (2) a post-operative survival time of more than one month. The obtained samples were immediately frozen after surgery. Overall survival (OS) time was defined as the time from the operation to the date of death or censored at the date of the last follow-up examination. The study end date was 31 March 2023. Commencing on May, 2012, the National Health Insurance Bureau of Taiwan broadened its coverage within the framework of health insurance benefits, encompassing the targeted drug bevacizumab's application in the treatment of adult patients experiencing relapsed glioblastoma multiforme. The administration of bevacizumab was typically synchronized with the emergence of disease progression, reflecting a commitment to transparency. This clinical determination was rooted in a thorough assessment of each patient's unique circumstances, alongside pertinent clinical variables. This systematic approach facilitated the precise deployment of bevacizumab, aligning its usage with the unmistakable signs of disease advancement. As a result, its focused deployment effectively managed the recurrence of glioblastoma. Nevertheless, it is imperative to acknowledge that certain individuals, prior to 2012, might not have received optimal counsel and treatment due to factors such as financial limitations or individual considerations.

### Endpoint

The primary endpoint of this study focused on overall survival (OS), which was defined as the period in months starting from the initiation of the first surgery and extending until the time of death. In cases where patients were still alive at the point of data censoring, the OS was calculated up to the date of the last follow-up. The secondary endpoints encompassed progression-free survival (PFS) and the assessment of adverse events. PFS, specifically, was defined as the duration in months commencing from the initiation of bevacizumab treatment and extending to the occurrence of disease progression or death. If patients were alive and had not encountered disease progression during data censoring, the calculated time interval was extended to the date of the last follow-up. These endpoint definitions were selected to comprehensively evaluate treatment outcomes and patient experiences.

### Genotyping assay

**Fig. 1:**
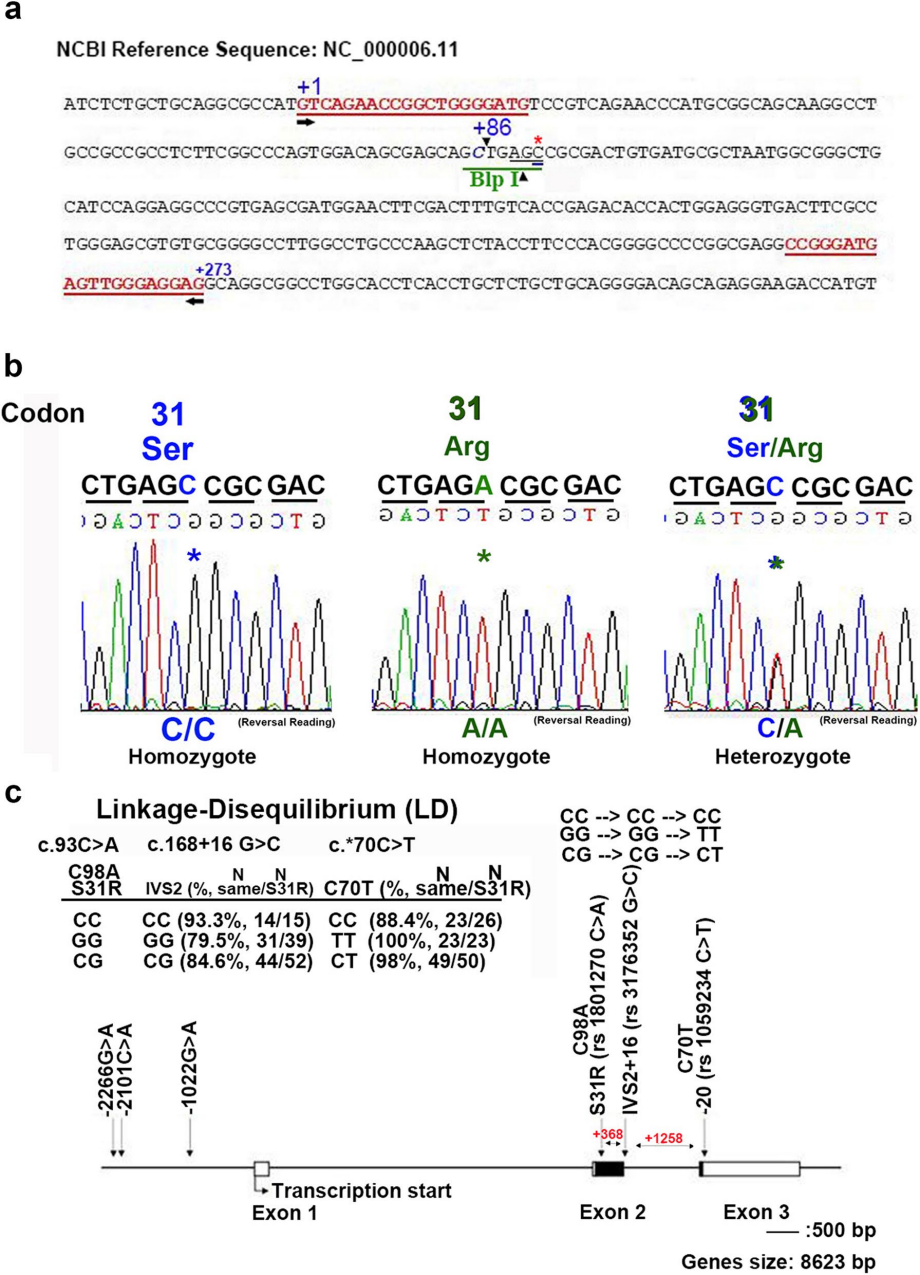
illustrates the schematic diagram of various CDKN1A polymorphisms. **a** Detailed sequences and location of CDKN1A c.93C > A (codon 31) in PCR production are presented. The NCBI association number is NC_000006.11. Red-colored words indicate the primers and green words represent the restriction enzyme Blp I cut site. The asterisk denotes the nucleotide of the CDKN1A c.93C > A polymorphism. **b** The PCR products of CDKN1A c.93C > A were analyzed by DNA sequencing, revealing three types of polymorphisms: Ser/Ser, Arg/Arg, and Ser/Arg, respectively. The asterisk represents the variant nucleotide of CDKN1A c.93C > A polymorphism. The heterozygote C/A genotyping of CDKN1A c.93C > A showed two signal peaks. **c** Schematic diagram of various CDKN1A polymorphisms, including S31R (rs 1801270C > A), IVS2 + 16 (rs 3176352G > C), and C70T (rs 1059234C > T). Red numbers indicate the number of nucleotides for each of these polymorphisms. After analyzing several samples using PCR–RFLP analysis, these three polymorphisms exhibit a high degree of linkage disequilibrium. For example, when S31R showed CC types (*n* = 15), IVS also exhibited CC types (93.3%, *n* = 14). Similarly, when S31R showed CC types (*n* = 26), C70T also displayed CC types (88.4%, *n* = 23). The pattern continues accordingly

**Fig. 2:**
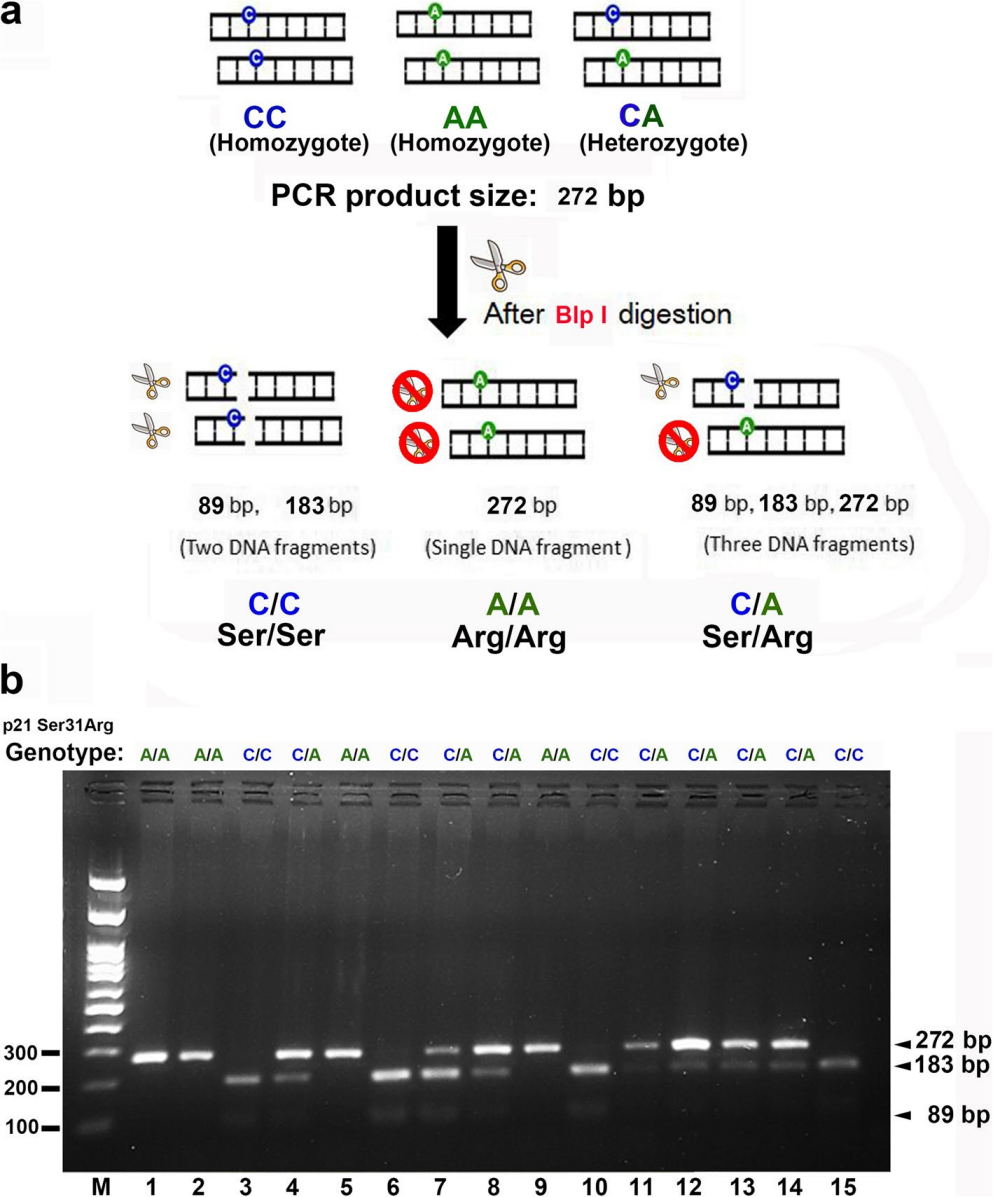
illustrates the Blp I PCR–RFLP analysis schematic diagram for the CDKN1A c.93C > A polymorphism. **a** The PCR products (272 bps) representing three types of CDKN1A c.93C > A polymorphism variants are shown: CC (homozygote), GG (homozygote), and CG (heterozygote). After digestion with the restriction enzyme Blp I, the CC genotype (Ser/Ser) is divided into two fragments (89 and 183 bps). The GG genotype (Arg/Arg) remains a single fragment of 272 bps due to the ineffectiveness of Blp I digestion. The CG genotype (Ser/Arg) results in three fragments (89, 183, and 272 bps) after Blp I digestion. **b** The Blp I PCR–RFLP analysis for the CDKN1A c.93C > A polymorphism is presented. M represents the DNA ladder. Lanes 1, 2, 5, and 9 show the Arg/Arg homozygotes, which are not cleaved by Blp I and display a 272-bp band. Lanes 3, 6, 10, and 15 represent the Ser/Ser homozygotes, which are cleaved by Blp I resulting in 183- and 89-bp bands. Lanes 4, 7, 8, 11, 12, 13, and 14 display the Ser/Arg heterozygotes with all three bands (272, 183, and 89 bp) after restriction digestion

We adhered to SNP names in accordance with the guidelines set forth by the Human Gene Nomenclature Committee (HGNC). Genomic DNA was extracted from frozen tumor tissues for the genotyping assay. The CDKN1A c.93C > A (codon 31) polymorphisms were analyzed using a polymerase chain reaction (PCR)—restriction fragment length polymorphism (RFLP) assay. PCR–RFLP analysis is a rapid and straightforward technique employed as an additional method to detect genetic polymorphisms in GBM. However, this method has certain limitations. The sequences of partial CDKN1A c.93C > A patients were determined using a DNA autosequencer (GeneAmp PCR System 2700 Thermal cycler; Applied Biosystems) (Fig. 1b). The primer sequence and PCR conditions for CDKN1A c.93C > A are described in Table 1 and Fig. 1a. For each sample, the amplified PCR product was digested with the Blp I restriction enzyme (New England Biolabs, Beverly, Massachusetts, USA). The digested reactions were incubated for 16 h at 37℃ [23]. Subsequently, the genotyping assay was conducted on a 2% agarose gel using molecular weight markers and visualized after staining with ethidium bromide (Fig. 2a). The Ser allele harbors a single Blp I restriction site (GCTNAGC), resulting in two fragments of 89 bp and 183 bp, while the Arg allele remains undigested, yielding a single band of 272 bp (Fig. 2b). Each genotyping assay included positive and negative controls, and 10% of the samples were randomly selected and run in duplicates, showing 100% concordance. The results were reproducible with no discrepancies in genotyping. The Supplementary Materials contain the provided DNA sequencing data. Moreover, the heterozygote C/A genotyping of CDKN1A c.93C > A exhibited two signal peaks in the DNA sequencing data (Fig. 1b), consistent with expectations. Additionally, we analyzed two other genotypes of CDKN1A polymorphisms (CDKN1A c.168 + 16G > C, rs3176352G > C, IV2 + 16; CDKN1A 3’UTR c.*70C > T, rs1059234C > T, C70T) and found them to be highly linked with S31R (CDKN1A c.93C > A; C98A, rs1801270) (Fig. 1c). Unprocessed images of the DNA electric gel are provided in the Supplementary Materials. Additionally, the Supplementary Materials provide detailed experimental procedures for Methylation-specific PCR and the identification of the IDH1 gene.

**Table 1:** Primers and Restriction Enzyme Used for Genotyping variants of p21 gene polymorphism

SNP	rs Number	Primer sequence	Denature, ℃/s	Annealing, ℃/s	Extension, ℃/s	PCR product	Restriction enzyme, ℃/min	SNP sequence	Allelic variants	DNA Frangment size
Ser31Arg	rs 1,801,270	F: 5'-gTCAgAACCggCTggggATg-3'	94℃/30 s	57℃/30 s	72℃/30 s	272 bp	Blp I, 37℃/120 min	C	Ser	89 + 183 bp
		R: 5'-CTCCTCCCAACTCATCCCgg-3'						A	Arg	272 bp
IVS2 + 16	rs 3,176,352	F: 5'-GACACCACTGGAGGGTGACT-3'	94℃/30 s	55℃/30 s	72℃/45 s	448 bp	Apal I, 37℃/120 min	C	Intron	159 + 289 bp
		R: 5'-GGTCTTTGCTGCCTACTTGC-3'						G	Intron	448 bp
C70T	rs 1,059,234	F: 5'-AGTTCTTCCTGTTCTCAGCAG-3'	94℃/30 s	60℃/30 s	72℃/30 s	327 bp	Pst I, 37℃/120 min	C	Intron	122 + 205 bp
		R: 5'-CCAGGGTATGTACATGAGGAG-3'						T	Intron	327 bp

*F* FORWARD primer, *R* Reverse primer, *bp* Base pairs

### Statistical analysis

Demographic data were presented as patient counts (percentages) for categorical variables and were compared using either the chi-squared test or Fisher's exact test. The overall survival (OS) outcomes were estimated using the Kaplan–Meier method, and differences in survival were assessed using the log-rank test. To investigate the association between CDKN1A and the 2-year overall survival (OS) of GBM patients, as well as the cumulative impact of CDKN1A SNP on the 2-year OS of GBM, adjusted hazard ratios (aHRs) with 95% confidence intervals (CIs) were employed. These aHRs were calculated using Cox proportional-hazards analyses and were adjusted for all the previously mentioned patient-level factors. Statistical analysis was conducted employing the Statistical Package for the Social Sciences (IBM SPSS version 22.0). All statistical analyses adhered to a two-sided approach, and significance levels of *p* < 0.05 and *p* < 0.01 were considered statistically significant.

## Results

### Demographic and clinicopathological characteristics of the participants

This retrospective study included 139 glioblastoma patients, consisting of 58 males and 81 females. The average age of the participants was 56 years (range: 20–92). There were no statistically significant differences in gender distribution among the different genotypes of CDKN1A c.93C > A (codon 31) (*p* = 0.888). At the end of the study, 80.6% of the patients had passed away, while 19.4% were still alive, with a median survival of 16.8 months. The clinical characteristics of the patients and the associations between the utilization of bevacizumab and various patient characteristics are outlined in Table 2. It is worth noting that patients over the age of 70 generally exhibited a poorer prognosis after surgery. The different variants of CDKN1A c.93C > A polymorphisms did not show any significant correlations with patient age, gender, tumor number, tumor size, tumor occurrence, or response to bevacizumab treatment.

**Table 2:** The Associations Between Bevacizumab Utilization and Diverse Patient Characteristics

	Patients (*n* = 139)		Bevacizumab				*p* value
			No used (*n* = 69)		Used (*n* = 70)		
	n	%	n	%	n	%	
**Age**							0.149
≦60	79	56.83%	35	50.72%	44	62.86%	
>60	60	43.17%	34	49.28%	26	37.14%	
**Gender**							0.943
Female	58	41.7%	29	42.03%	29	41.43%	
Male	81	58.3%	40	57.97%	41	58.57%	
**Tumor number**							0.849
Solitary	116	83.45%	58	84.06%	58	82.86%	
Multiple	23	16.55%	11	15.94%	12	17.14%	
**Tumor resection size**							0.604
≦3 cm	20	14.39%	11	15.94%	9	12.86%	
> 3 cm	119	85.61%	58	84.06%	61	87.14%	
**Tumor occurrence**							0.969
Primary	115	82.73%	57	82.61%	58	82.86%	
Recurrence	24	17.27%	12	17.39%	12	17.14%	
**Codon 31 (CDKN1A)**							0.888
CC	32	23.02%	17	24.64%	15	21.43%	
AA	38	27.34%	18	26.09%	20	28.57%	
CA	69	49.64%	34	49.28%	35	50.00%	

Chi-square test. *p < 0.05, **p < 0.01. Statistical significance

### Genotyping

Association between CDKN1A c.93C > A Polymorphism and Glioblastoma Risk.

Tables 2 and 3 present the frequencies of genotypes and alleles within the CDKN1A gene. In our glioblastoma cases, we observed frequencies of 23.02% (32/139) for Ser/Ser, 27.34% (38/139) for Arg/Arg, and 49.64% (69/139) for Ser/Arg genotypes. Notably, all observed results were found to conform to the principles of the Hardy–Weinberg equilibrium. Furthermore, an assessment of the relationship between the CDKN1A c.93C > A genotype and demographic as well as clinicopathological characteristics of GBM patients was conducted, encompassing factors such as IDH1 gene status and MGMT promoter methylation status (as demonstrated in Table 3). Tables 2 and 3 provide additional insight by demonstrating that specific potential risk factors associated with GBM, including age, gender, tumor count, tumor resection size, IDH1 gene status, and MGMT gene methylation status, did not exhibit significant associations with the CDKN1A genotype. We investigated the link between individual CDKN1A c.93C > A genotypes and their correlation with the 2-year overall survival (OS) among GBM patients. This evaluation was conducted using Cox proportional-hazards models, which were adjusted for variables such as age, gender, stage, and the usage of bevacizumab, as illustrated in Table 4. However, in both univariate and multivariate analyses, none of the computed hazard ratios achieved statistical significance.

**Table 3:** Clinical characteristics of GBM patients and CDKN1A genotype (c.93C > A)

	codon 31 (c.93C > A)									
	CC (*n* = 32)		AA (*n* = 38)		CA (*n* = 69)		*p* value	AA + CA (*n* = 107)		*p* value
	n	%	n	%	n	%		n	%	
**Age**							0.104			0.253
≦60	21	65.63%	25	65.79%	33	47.83%		58	54.21%	
>60	11	34.38%	13	34.21%	36	52.17%		49	45.79%	
**Gender**							0.114			0.136
Female	17	53.13%	11	28.95%	30	43.48%		41	38.32%	
Male	15	46.88%	27	71.05%	39	56.52%		66	61.68%	
**Tumor number**							0.194			0.074
Solitary	30	93.75%	30	78.95%	56	81.16%		86	80.37%	
Multiple	2	6.25%	8	21.05%	13	18.84%		21	19.63%	
**Tumor resection size**							0.346			0.566
≦3 cm	3	9.38%	8	21.05%	9	13.04%		17	15.89%	
> 3 cm	29	90.63%	30	78.95%	60	86.96%		90	84.11%	
**Tumor occurrence**							0.888			0.780
Primary	27	84.38%	32	84.21%	56	81.16%		88	82.24%	
Recurrence	5	15.63%	6	15.79%	13	18.84%		19	17.76%	
**IDH1 gene status**							0.741			0.582
Wild type	26	81.25%	28	73.68%	54	78.26%		82	76.64%	
Mutation	6	18.75%	10	26.32%	15	21.74%		25	23.36%	
**MGMT promoter status**							0.789			0.986
Uumethylation	18	56.25%	23	60.53%	37	53.62%		60	56.07%	
Methylation	14	43.75%	15	39.47%	32	46.38%		47	43.93%	

Chi-square test. *p < 0.05, **p < 0.01. Statistical significance

**Table 4:** Relationship between CDKN1A codon 31 SNP and 2-year overall survival of GBM patients

	No. of subjects	No. of cases (%)		**Univariate**				**Multivariable**^a^			
				HR	95%CI		*p*	HR	95%CI		*p*
Codon 31											
CC	32	18	(22.5%)	Reference				Reference			
AA	38	18	(22.5%)	0.78	(0.41-	1.51)	0.467	0.77	(0.40-	1.51)	0.453
CA	69	44	(55.0%)	1.22	(0.70-	2.11)	0.480	1.26	(0.72-	2.22)	0.425

Cox proportional hazard regression. *p < 0.05, **p < 0.01

^a^Adjusted for age, gender and bevacizumab. *HR* Hazard ratio

### Genotype effects on overall survival after bevacizumab treatment

**Fig. 3:**
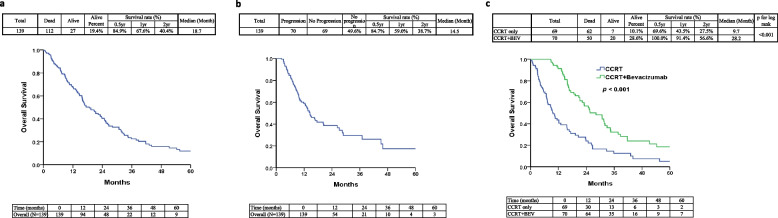
Kaplan–Meier curves depicting overall survival (OS) and Progression-Free Survival (PFS) in patients receiving CCRT and CCRT plus bevacizumab. **a** Overall survival duration for the entire patient cohort. **b** PFS in patients with glioblastoma treated with bevacizumab. **c** Kaplan–Meier curves illustrating overall survival in patients receiving standard CCRT treatment and CCRT plus bevacizumab treatment. ** indicates *p* < 0.001

The study encompassed the enrollment of 139 GBM patients, with a median follow-up duration spanning 18.7 months (as visually represented in Fig. 3a). Our investigation ventured further into a meticulous assessment of the implications associated with the amalgamation of bevacizumab and chemotherapy concerning the outcomes of progression-free survival (PFS) and overall survival (OS) within the cohort of GBM patients. Within our study, we operationalized "PFS" as the timeframe commencing from the commencement of bevacizumab treatment and extending to the occurrence of disease progression or mortality. This particular definition was meticulously selected to exclusively probe the influence of bevacizumab treatment on the twin facets of disease progression and survival outcomes, with an explicit focus on the temporal period post-administration of the treatment. Although the median PFS duration within the subset of patients subjected to CCRT plus bevacizumab treatment did not manifest a statistically significant expansion (as visually indicated in Fig. 3b) (median of 14.5 months), our scrutiny of OS outcomes divulged a notably constructive impact. This was evident in the comparative evaluation between individuals who exclusively underwent chemotherapy (*N* = 69) and those who underwent a combined therapeutic regimen encompassing bevacizumab (*N* = 70), as vividly depicted in Fig. 3c.

Statistical analysis demonstrated a significant improvement (log-rank *p* < 0.001) in median OS from 9.7 to 28.2 months for the CCRT plus bevacizumab group compared to the CCRT group (Fig. 3c). These findings from the retrospective study strongly suggest that bevacizumab can extend the OS of patients with recurrent GBM [24].

**Fig. 4:**
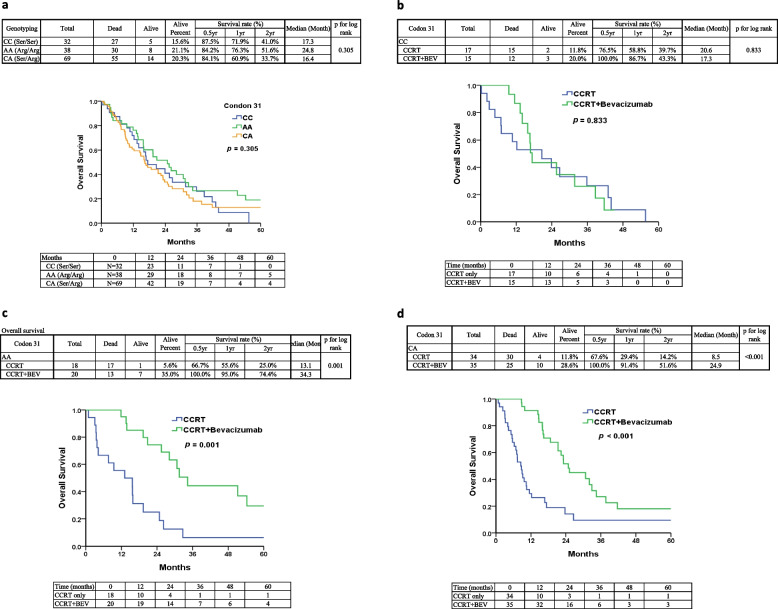
Genotypes of CDKN1A c.93C > A variants and Kaplan–Meier plots of overall survival (OS) for three groups of GBM patients. **a** Survival curves for GBM patients stratified by CDKN1A c.93C > A genotypes. **b** Comparison of estimated OS in patients with CDKN1A c.93C > A Ser/Ser genotypes between those treated with CCRT and those treated with CCRT plus bevacizumab. **c** Estimated OS in patients with CDKN1A c.93C > A Arg/Arg genotypes for CCRT and CCRT plus bevacizumab treatment. **d** Estimated OS in patients with CDKN1A c.93C > A heterozygous Ser/Arg genotypes for CCRT and CCRT plus bevacizumab treatment. ** indicates *p* < 0.001

To further explore the relationship between the CDKN1A c.93C > A polymorphism and GBM, we analyzed the overall survival data and genotyping information of all GBM patients. Using the log-rank test and Kaplan–Meier survival curve analysis, we found that the OS analysis comparing the CDKN1A c.93C > A variants did not yield significant results (Fig. 4a). However, a slightly better survival rate was observed in patients with the AA (Arg/Arg) variant.

We also investigated the impact of combining bevacizumab with chemotherapy on the overall survival (OS) of GBM patients with different CDKN1A genotypes. As shown in Fig. 4b, patients with the CC (Ser/Ser) genotype who received CCRT plus bevacizumab had a median survival of 17.3 months, similar to those with CCRT alone (*p* = 0.833). Interestingly, in contrast, patients with the AA (Arg/Arg) and CA (Ser/Arg) genotypes exhibited significantly longer median survival when treated with CCRT plus bevacizumab (34.3 and 24.9 months, respectively) compared to CCRT alone (13.1 and 8.5 months, respectively) (*p* = 0.001 and *p* < 0.001, respectively, Fig. 4c and d). Furthermore, patients with the CC genotype who received CCRT alone exhibited a higher median survival compared to the other two genotypes. Overall, GBM patients with the AA (Arg/Arg) and CA (Ser/Arg) genotypes demonstrated significantly prolonged survival in the CCRT plus bevacizumab treatment group compared to those with the CC (Ser/Ser) genotype (Fig. 4).

These findings suggest that GBM patients with the AA (Arg/Arg) and CA (Ser/Arg) genotypes of CDKN1A c.93C > A have significantly longer overall survival intervals when treated with CCRT plus bevacizumab compared to those with the CC (Ser/Ser) genotype in the same treatment group.

We broadened our inquiry to delve into the relationship between CDKN1A c.93C > A and the 2-year overall survival, stratifying the data according to the methylation status of the MGMT promoter and the IDH1 gene status (as illustrated in Table 5). Despite conducting both univariate and multivariate analyses, none of the calculated hazard ratios achieved statistical significance. Finally, we conducted a comprehensive risk assessment to determine the potential survival benefits associated with the utilization of BEV. Drawing from our research results, we employed the CDKN1A SNP, IDH1 gene status, and MGMT promoter methylation level to categorize risks. A summary of these findings is presented in Table 6. Among GBM patients with the CDKN1A c.93C > A genotype polymorphism, both univariate and multivariate analyses unveiled a substantial escalation in the risk of mortality for individuals with AA or CA genotypes who did not use BEV. In contrast, patients with the CC genotype exhibited no notable association with BEV usage. A comparable scenario is also evident within the MGMT methylation and IDH1 gene mutation groups: irrespective of MGMT methylation or IDH1 mutation status, individuals who refrain from BEV usage face a notably elevated risk of mortality compared to patients who undergo BEV treatment (Table 6). The aforementioned findings collectively suggest that the utilization of BEV appears to confer survival advantages to GBM patients, with the exception of those with the CDKN1A c.93C > A CC (Se/Ser) genotype.

**Table 5:** The stratified impact of MGMT gene promoter region methylation, IDH1 gene status, and CDKN1A c.93C > A genotype on the 2-year overall survival of GBM patients

	No. of subjects	No. of cases (%)		**Univariate**				**Multivariable**^a^			
				HR	95%CI		*p*	HR	95%CI		*p*
CC vs CA + AA											
IDH1 gene status											
Wild type	26	15	(83.3%)	0.99	(0.55-	1.77)	0.968	1.04	(0.57-	1.91)	0.890
Mutation	6	3	(16.7%)	1.30	(0.38-	4.50)	0.679	1.29	(0.33-	5.05)	0.714
MGMT promoter status											
Unmethylation	18	8	(44.4%)	1.62	(0.75-	3.48)	0.218	1.85	(0.84-	4.06)	0.127
Methylation	14	10	(55.6%)	0.66	(0.31-	1.37)	0.260	0.52	(0.24-	1.13)	0.098

Cox proportional hazard regression. *p < 0.05, **p < 0.01

^a^Adjusted for age, gender and bevacizumab. *HR* Hazard ratio

**Table 6:** The stratified influence of CDKN1A c.93C > A genotype, MGMT gene promoter region methylation, IDH1 gene status, and the utilization of Bevacizumab on the 2-year overall survival of GBM patients

	No. of subjects	No. of cases (%)		**Univariate**				**Multivariable**^a^			
				HR	95%CI		*p*	HR	95%CI		*p*
Bev (-) vs Bev ( +)											
Codon 31											
CC	17	10	(19.6%)	1.36	(0.54-	3.47)	0.516	2.08	(0.75-	5.74)	0.157
AA	18	13	(25.5%)	5.12	(1.80-	14.53)	0.002**	5.87	(1.94-	17.80)	0.002**
CA	34	28	(54.9%)	4.07	(2.17-	7.64)	** < 0.001**	4.57	(2.36-	8.82)	** < 0.001**
IDH1 gene status											
Wild type	53	39	(76.5%)	3.10	(1.84-	5.20)	** < 0.001**	4.19	(2.43-	7.22)	** < 0.001**
Mutation	16	12	(23.5%)	3.84	(1.41-	10.40)	0.008**	3.97	(1.36-	11.62)	0.012*
MGMT promoter status											
Unmethylation	40	29	(56.9%)	2.90	(1.57-	5.37)	0.001**	3.43	(1.83-	6.42)	** < 0.001**
Methylation	29	22	(43.1%)	3.76	(1.87-	7.56)	** < 0.001**	4.24	(2.06-	8.72)	** < 0.001**

Cox proportional hazard regression. *p < 0.05, **p < 0.01. Bold indicates a statiscally significant difference with a *p*-value less than 0.05

^a^Adjusted for age, sex, Tumor resection size and Tumor occurrence. Bev (-): bevacizumab used; Bev ( +): bevacizumab not used; *HR* Hazard ratio

**Fig. 5:**
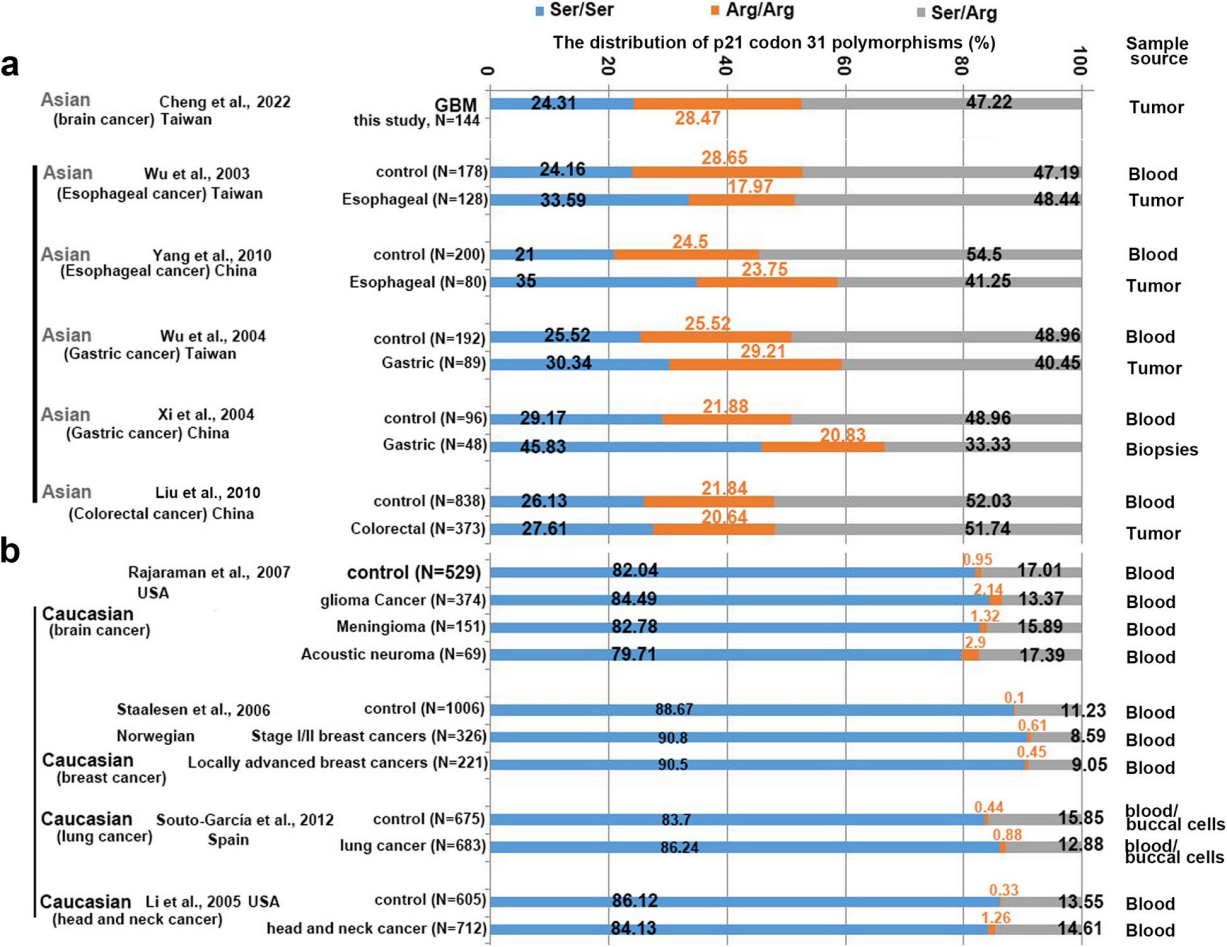
illustrates the comparison of CDKN1A c.93C > A polymorphism distributions in normal control groups from various case/control studies. Panel **a** shows the distribution of cancer cases from across Asia population (ref. [19, 25–28]), while panel **b** presents the distribution in the Caucasian population (ref. [17, 29–31]). The analyzed cancer cases demonstrated a similar distribution to control groups in other studies involving Caucasians (from the USA, Spain, and Europe). Notably, studies conducted on the Chinese population revealed a distinct allele distribution, characterized by a higher frequency of the Arg allele and Ser/Arg heterozygotes compared to Caucasians. N represents the number of analyzed cases

This study exclusively involved GBM patients in Taiwan, making it particularly relevant to individuals with Asian heritage and nationality. To comprehensively explore the diversity of CDKN1A c.93C > A polymorphisms across different ethnicities, we conducted an extensive review of pertinent literature within the Asian population (references [19, 25–28]) and the Caucasian population documented in the PubMed database (references [17, 29–31]) (as illustrated in Fig. 5). While our study did not include a healthy control group, we meticulously analyzed relevant studies that provided accessible CDKN1A c.93C > A data in Asians, even if derived from different sources. Notably, these analytical samples were drawn from a variety of studies, albeit without distinguishing among different disease types.

As depicted in Fig. 5a, the distribution range for the Ser/Ser genotype of CDKN1A c.93C > A in the Asian population ranged from 21% to 45.83% (blue color), while the Arg/Arg and Arg/Ser genotypes had ranges of 17.97% to 29.21% (orange) and 33.33% to 54.5% (gray), respectively. Our data align with the expected distribution of CDKN1A c.93C > A genotypes in the Asian population (Fig. 5a, lane 1). Interestingly, among the Caucasian population (Fig. 5b, blue color), the distribution range for the Ser/Ser genotype of CDKN1A c.93C > A extends from 79.71% to 90.8%. Additionally, the distribution of the Ser/Arg genotype (gray color) ranged from 8.59% to 17.39%. The Arg/Arg genotype (orange color) was rarely found in the Caucasian population, with a distribution range of 0.1% to 1.26% (Fig. 5b). These findings highlight substantial differences in the distribution of CDKN1A c.93C > A genotypes between the Asian and Caucasian populations. However, it is important to note that these data alone do not establish CDKN1A c.93C > A as a risk factor for glioma.

## Discussion

This study focuses on the distribution of CDKN1A c.93C > A (codon 31) polymorphisms in GBM patients and explores the impact of CCRT plus bevacizumab treatment on specific ethnic groups. Our findings regarding the distribution of CDKN1A codon genotypes in the Taiwanese population of GBM patients revealed the following frequencies: Ser/Ser (23.02%; 32/139), Arg/Arg (27.34%; 38/139), and Ser/Arg (49.64%; 69/139) genotypes. However, none of these genotypes were directly associated with the overall survival of GBM patients. On the other hand, our evaluation of the effect of bevacizumab on the OS of GBM patients demonstrated that it prolongs the overall survival of patients with recurrent GBM.

Interestingly, when analyzing the different genotypes of CDKN1A c.93C > A polymorphisms, we observed a significant association between the Arg/Arg homozygous and Ser/Arg heterozygous genotypes and prolonged overall survival compared to the Ser/Ser genotype group. While these results are promising, they should be further validated through well-designed prospective randomized control trials.

The rising incidence and mortality of GBM in Taiwan have prompted us to conduct this retrospective study, aiming to investigate the prevalence and association between these SNPs and GBM development in the Taiwanese population. Currently, treatment options for recurrent glioblastoma are limited, and their efficacy remains uncertain. Standard chemotherapy protocols for recurrent glioblastoma are yet to be established. Given the highly vascular nature of GBM, antiangiogenic agents have been widely utilized in the treatment of recurrent glioblastoma. Bevacizumab (BEV), known for its inhibitory effect on upstream mediators of tumor angiogenesis, has been proposed as a therapeutic option for glioblastoma. Recent research has demonstrated that combination treatment with BEV induces significant transcriptional changes that impact glioblastoma [32]. In Taiwan, bevacizumab has achieved certain success in the treatment of various cancers, including breast cancer [33], colorectal cancer [34–37], non-small cell lung cancer [38], and liver cancer [39]. According to clinical trials and case reports, bevacizumab has been shown to prolong progression-free survival (PFS), reduce tumor size, improve quality of life, and extend overall survival (OS).

The presence of neo-angiogenesis in GBM indicates the potential effectiveness of anti-angiogenic therapies. Therefore, the use of bevacizumab therapy has shown promising outcomes in terms of progression-free survival in recurrent GBM [40–42]. Several clinical trials have been initiated to investigate the impact of combination therapy with BEV, and while such therapy significantly improves PFS, there is sufficient evidence to support the prolongation of overall survival. However, our retrospective study demonstrated that recurrent GBM patients treated with BEV exhibited a significant benefit in terms of PFS and a trend towards improved OS (CCRT plus TMZ vs. CCRT plus TMZ + BEV median PFS: 14.5 months; median OS: 18.7 months) [24]. Intriguingly, various clinical data have confirmed that BEV demonstrates clinically meaningful efficacy and an acceptable safety profile not only in Asian populations but also the global populations [43–45], which is consistent with our findings. We speculated the observed benefits of BEV treatment may be attributed to factors such as low-dose BEV (< 10 mg/kg) or extending the treatment period until recurrence occurs. In fact, a retrospective study has suggested that lower doses of BEV (< 3 mg/kg/week) may be more effective and associated with fewer adverse events in GBM treatment [46]. Nevertheless, it is important to acknowledge that the small sample size in our study may limit the generalizability of the results.

There is growing evidence suggesting the involvement of CDKN1A expression in various malignancies, such as tonsillar [47], gastric [48], lung [49], and brain [50] cancers. However, the results from studies investigating the role of CDKN1A expression are inconsistent. Different studies have reported contradictory findings, suggesting that CDKN1A can either promote or inhibit apoptosis and differentiation [51–53]. For instance, high CDKN1A expression has been associated with a favorable response to chemotherapy in esophageal cancer [54], while Koopmann et al. found no involvement of CDKN1A mutation in brain tumor formation [55]. Despite the mixed results, several reports have explored the relationship between CDKN1A polymorphisms and cancer risks, although no definite conclusions have been reached. For example, the CDKN1A 3’UTR c.*70C > T polymorphism is believed to cause a functional change in CDKN1A. This polymorphism affects messenger RNA stability in a crucial region for cell differentiation and may increase cancer risk by altering proliferation [56, 57]. Likewise, the CDKN1A c.168 + 16G > C variant, formerly denoted as p21 rs3176352 G/C (IVS2 + 16 G.C), is situated within intron 2 of the CDKN1A gene, positioned 16 base pairs downstream from the splicing site. This C to G is predicted to impact CDKN1A mRNA splicing [27]. Furthermore, a study that reported the CDKN1A Arg allele (rs1801270, S31R) is associated with lower expression of the downstream target gene of CDKN1A [58]. Interestingly, our analysis revealed that two CDKN1A polymorphisms, c.168 + 16G > C and 3’UTR c.*70C > T, appeared to be in linkage disequilibrium with Ser31Arg (CDKN1A c.93C > A) in the Chinese population, which aligns with the finding of Choi et al., in a Korean population [59]. Combined analysis of these three CDKN1A polymorphisms may offer better predictive value for tumorigenesis risk compared to analyzing a single polymorphism alone.

In our study, we duly recognize the significance of accounting for the origin of SNP data when engaging in cross-population result comparisons. We have thoughtfully incorporated SNP data from diverse populations into our analysis and discussion, thus furnishing a comprehensive contextual framework for our findings. It is imperative to underscore that the data from other populations may have originated from blood samples, whereas our study harnessed tumor DNA for analysis. One pertinent aspect to consider while utilizing tumor DNA lies in the potential for mutations at polymorphic sites, a phenomenon often attributed to the genomic instability inherent in cancer. Tumor DNA has the propensity to accumulate genetic alterations that could conceivably impact the precision of SNP genotyping. This occurrence could potentially introduce variances in allele frequencies, thereby influencing the interpretation of results, particularly when compared against data originating from non-tumor DNA sources.

Genetic polymorphism frequencies frequently exhibit variations among ethnic groups, suggesting potential ethnic and tumor-specific disparities in the cancer susceptibility associated with CDKN1A c.93C > A polymorphisms. However, it's important to note that differences in genotype distribution may not necessarily indicate fundamental disparities in the underlying mechanisms governing the pathogenesis and initiation of GBM across distinct populations. Our data regarding the distribution of genotypes in CDKN1A c.93C > A showed various shared and unshared GBM characteristics between Taiwanese and Caucasian patients. Our data showed that the total percentage number in groups of Arg/Arg and Ser/Arg of CDKN1A c.93C > A is 75.69%, and this population of genotyping obtained the benefit of overall survival after bevacizumab. Interestingly, the occurrence of the genotypes mentioned above in the Caucasian population is below 20%, with over 80% belonging to gene groups carrying the CC genotype (Ser/Ser). Consequently, the potential advantageous impact of bevacizumab may not hold significant significance within the Caucasian demographic. Furthermore, the overall survival (OS) benefit of bevacizumab in brain cancer treatment remains uncertain, as certain studies have not demonstrated a significant extension in OS. Although our data showed that these SNPs may not be potential markers for the prediction of GBM, such polymorphisms may have an influence on GBM susceptibility in combination with certain other elements, then affect the survival of GBM patients. Furthermore, many clinical trials have shown the efficacy of bevacizumab in treating malignancies and an acceptable safety profile in Asian populations, as well as in global populations [24, 44, 45], suggesting our results are consistent with previous report data. However, it should be noted that the treatment effectiveness of bevacizumab may vary among individuals and can be influenced by various factors, including tumor characteristics, patient's physical condition, and combination with other treatment modalities. Therefore, before using bevacizumab or any other medication, patients should engage in detailed discussions and evaluations with their doctors to determine the optimal treatment plan.

The available data suggest that the S31R polymorphism in the p21 gene may serve as a predictive marker for improved overall survival in patients undergoing bevacizumab treatment. However, it is essential to acknowledge that this conclusion remains speculative and lacks direct data support. When considering potential molecular mechanisms for this observation, several points are worth considering. Firstly, the polymorphism could impact cell cycle regulation since p21 plays a vital negative regulatory role during the G1/S and G2/M transitions of the cell cycle. Any functional or stability changes in the p21 protein due to the polymorphism might disrupt cell cycle regulation, potentially influencing tumor cell growth and proliferation. Secondly, polymorphism may also influence DNA damage repair pathways, as p21 is involved in cellular responses and DNA damage repair. The altered functionality of p21 due to the polymorphism could lead to changes in reactions to therapy-induced DNA damage, potentially affecting treatment effectiveness and patient survival. Lastly, considering bevacizumab's mechanism of action as an anti-angiogenic drug that inhibits VEGF activity to obstruct tumor blood supply, the p21 protein could be associated with angiogenesis inhibition. The polymorphism might affect the regulation of the VEGF pathway, potentially impacting the efficacy of bevacizumab treatment. It is important to reiterate that these speculations are based on limited evidence, and further research and clinical validation are necessary to confirm the validity of this conclusion and unravel the underlying molecular mechanisms.

## Conclusion

In conclusion, our data suggest that the CDKN1A c.93C > A, S31R polymorphism may serve as a predictive marker for improved overall survival in patients undergoing bevacizumab treatment. Although our sample size is relatively small, these findings indicate a potential association between the Arg/Arg and Ser/Arg genotypes of the CDKN1A c.93C > A polymorphism and the beneficial effect of bevacizumab in glioblastoma treatment. However, further confirmation of these findings is warranted through additional larger studies and tissue-specific biological characterization.

## Availability of data and materials

The data that support the findings of this study are available from Taichung Veterans General Hospital but restrictions apply to the availability of these data, which were used under license for the current study, and so are not publicly available. Data are however available from the authors upon reasonable request and with permission of Taichung Veterans General Hospital.

### Supplementary Information

**Additional file 1:** **Supplementary Figure 1.** showcases the unaltered and unprocessed versions of Figure 2b. Importantly, no adjustments were made to the exposure parameters of this image. **Supplementary Data.** **Supplementary Table 1.** Primer sequences, annealing temperature and product size for methylation-specific polymerase of MGMT gene.

## Abbreviations

CDKN1A: Cyclin-Dependent Kinase Inhibitor 1A
PCR–RFLP: Polymerase chain reaction-restriction fragment length polymorphism; Cip1:CDK-interacting protein 1
WAF1: Wild-type p53-activated fragment 1
GBM: Glioblastoma multiforme
SNPs: Single-nucleotide polymorphisms
CI: Confidence interval
DNA: Deoxyribonucleic acid
MRI: Magnetic resonance imaging
OS: Overall survival
PFS: Progression-free survival
SD: Standard deviation
BEV: Bevacizumab
VEGF: Vascular endothelial growth factor
VEGFR: Vascular endothelial growth factor receptor
TCGA: The Cancer Genome Atlas
MGMT: O-6-methylguanine-DNA methyltransferase
IDH1: Isocitrate dehydrogenase 1
HGNC: The Human Gene Nomenclature Committee
CCRT: Concurrent Chemoradiotherapy
TMZ: Temozolomide

## Declarations

Ethics approval and consent to participate

The study protocols received approval from the Medical Ethics Committee of Taichung Veterans General Hospital (Approval number: CF17263B-4). Informed consent was obtained from all subjects or their legal guardians prior to surgery, and the collected samples were promptly frozen. The validation cohort consisted of 254 cases selected from the primary cohort based on the following criteria: (1) availability of follow-up data and samples, and (2) postoperative survival time exceeding 1 month. Between January 2010 and August 2022, tissue specimens were collected from 139 patients diagnosed with GBM. Exclusion criteria included: 1. GBM patients with unconfirmed pathology, 2. GBM patients with spinal involvement, and 3. GBM patients with incomplete data records. All methods strictly adhered to the guidelines and regulations of Taichung Veterans General Hospital and were conducted in accordance with the Declaration of Helsinki.
