# Supplementary material for: Polymorphism at codon 31 of CDKN1A (p21) as a predictive factor for bevacizumab therapy in glioblastoma multiforme
Source: BMC Cancer. 2023 Sep 20;23:886. doi: 10.1186/s12885-023-11400-5 (PMC10510274; doi:10.1186/s12885-023-11400-5)
Supplement: Supplementary file 1 — Additional file 1: Supplementary Figure 1. showcases the unaltered and unprocessed versions of Figure 2b. Importantly, no adjustments were made to the exposure parameters of this image. Supplementary Data. Supplementary Table 1. Primer sequences, annealing temperature and product size for methylation-specific polymerase of MGMT gene. [file 12885_2023_11400_MOESM1_ESM.doc]

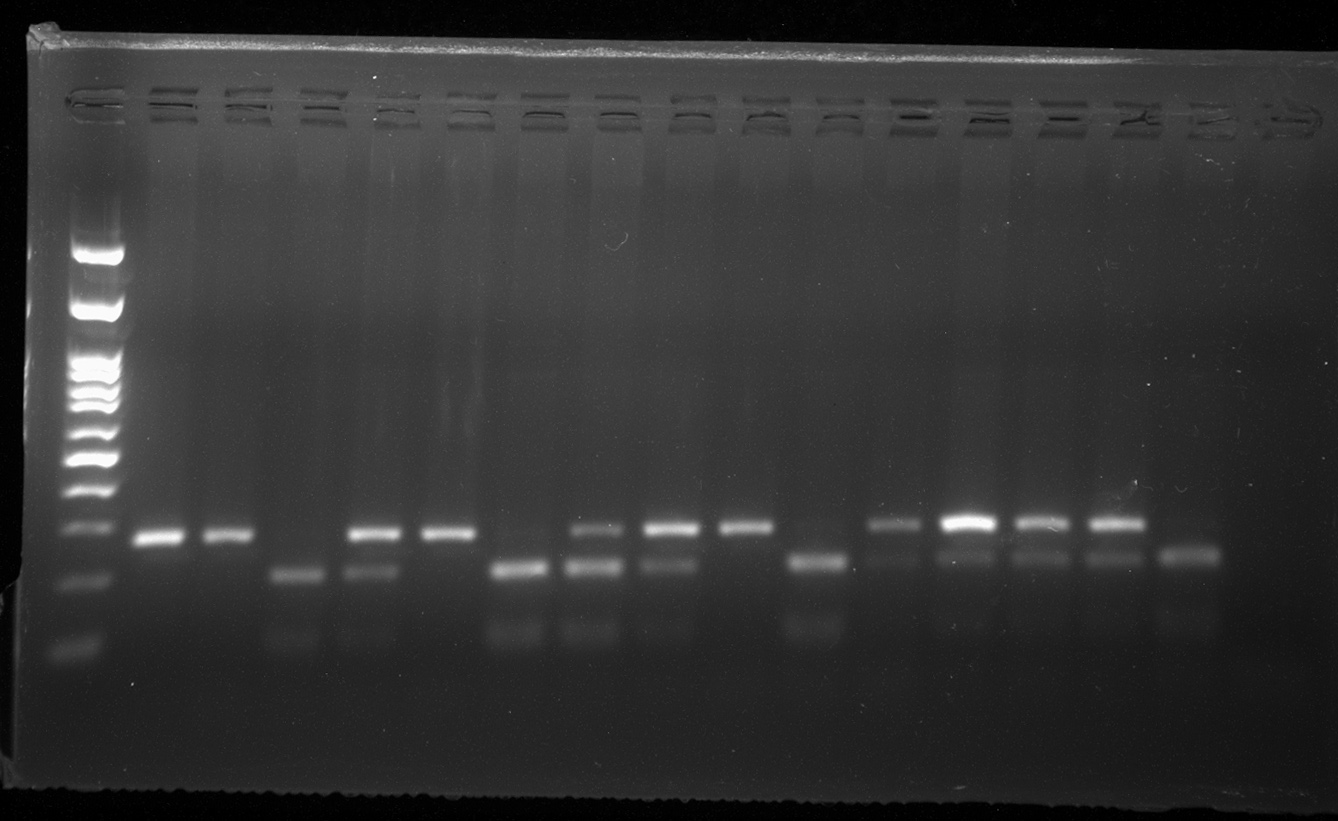


Supplementary Figure 1 showcases the unaltered and unprocessed versions of Figure 2b. Importantly, no adjustments were made to the exposure parameters of this image.

**Supplementary Data**

We provide sequencing results for the three genotypes (CC, AA, and CA) associated with p21 codon 31. The corresponding additional files (CC-GBM145_P21-codon31-R.ab1, AA-PO177_P21-codon31-R.ab1, and CA-GBM285_P21-codon31-R.ab1) should be opened using the Chromas.exe program. All files are compressed in the Chromas.rar archive. Gene readout is conducted using the reverse primer.

Comprehensive Assessment of Progression-Free Survival (PFS) through Clinical Evaluations and MRI Scans in Study Participants

The evaluation of Progression-Free Survival (PFS) encompassed a multi-faceted approach, integrating both clinical assessments and MRI scans. Regular clinical evaluations diligently tracked patients' symptoms and holistic well-being. Additionally, scheduled MRI scans were meticulously conducted at specified intervals, facilitating visual scrutiny of potential alterations in tumor dimensions and attributes. A holistic PFS evaluation was attained across the study cohort by synergizing clinical appraisals with radiological imaging. This methodology ensured a comprehensive and accurate assessment of treatment efficacy and disease progression.

**Rationale for Choosing PCR-RFLP over Allelic Discrimination Assay in Realtime PCR for Genetic Variation Analysis**

Authors opted for the use of PCR-RFLP over a cost-effective technique like allelic discrimination assay in realtime PCR for several reasons. PCR-RFLP has been widely established and utilized in various genetic studies, offering a proven method for detecting specific DNA variations. It involves enzymatic digestion of amplified DNA, allowing for the identification of target polymorphisms.

While allelic discrimination assay in realtime PCR is indeed a sensitive method, its implementation often requires specialized equipment and reagents, potentially increasing the overall cost and complexity of the experimental setup. In contrast, PCR-RFLP utilizes conventional PCR equipment and is generally more accessible in terms of laboratory resources.

Furthermore, the decision to use PCR-RFLP might have been influenced by factors such as the specific objectives of the study, the availability of expertise, and the familiarity of the research team with the chosen methodology. Overall, the authors chose PCR-RFLP as a practical and effective alternative to investigate the desired genetic variations in a cost-efficient manner.

**The utilization of two distinct cutoff levels for p-values serves specific analytical objectives within this study.**

The lower threshold, set at p < 0.05, embodies a conventional standard widely employed in statistical analysis to ascertain the statistical significance of outcomes. This criterion aids in determining whether an observed effect or correlation is likely to be genuine or if it could have arisen purely by chance.

On the other hand, the higher cutoff level, denoted as p < 0.01, adopts a more stringent stance, signifying an elevated level of significance. This criterion is enlisted when researchers seek to minimize the risk of committing a Type I error (false positive) and demand more compelling evidence to establish a notable outcome. This level of significance is often chosen in scenarios where pivotal findings are at stake or when the research context mandates a heightened level of confidence in the detected associations.

By incorporating both these cutoff levels, the study endeavors to provide a comprehensive evaluation of statistical significance, while accommodating diverse degrees of confidence and potential error rates. This dual-tiered approach facilitates a more nuanced interpretation of the results and augments the overall robustness of the findings.

**Mutation analysis of IDH1 gene**

Exon 4 of IDH1 harbors single nucleotide polymorphisms (SNPs) at positions V71, G97, G123, and R132. Amplification was achieved utilizing forward primers 5'-AggTggTCCTTAgACTTACAC-3' and reverse primers 5'-CAgTTATACATATgCATTTCTC' for each specific position. The IDH1 mutations were evaluated via direct sequencing of the polymerase chain reaction (PCR) product, employing the previously delineated methodology [1].

**Methylation-specific-PCR assay**

Methylation-Specific PCR (MS-PCR) was utilized to evaluate the DNA methylation status within the promoter regions of MGMT. This approach was carried out following the methodology outlined in a previous study [2]. In each reaction, 1.2 μL of both forward and reverse primers were combined with 12.5 μL of HotStart Taq Premix from RBC Bioscience, along with bisulfite-converted DNA as per the manufacturer's instructions. Detailed information regarding primer sequences, individual annealing temperatures, and resulting product sizes from the MS-PCR are provided in Supplementary Table 1.

Our MS-PCR protocol closely mirrored the methodology described in the earlier study [2]: a preliminary denaturation step at 95 °C for 10 minutes, succeeded by 35 cycles of denaturation at 95 °C for 30 seconds, annealing for 30 seconds, and extension at 72 °C for 48 seconds. A final extension step at 72 °C for 4 minutes marked the conclusion of the amplification process. Following amplification, the MS-PCR products were combined with a loading buffer, subjected to electrophoresis on a 2% agarose gel for 25 minutes, and subsequently visualized using an ultraviolet transilluminator after being stained with 0.2 μL of gel dye.

Supplementary Table 1. Primer sequences, annealing temperature and product size for methylation-specific polymerase of MGMT gene.

| Gene name |  | Forward primer (5' --> 3') | | Annealing temperature (℃) | | | Product size (bp) | |
| --- | --- | --- | --- | --- | --- | --- | --- | --- |
| MGMT | M | F: TTTCgACgTTCgTAggTTTTCgC |  | 53 |  |  | 81 |  |
|  |  | R: gCACTCTTCCgAAAACgAAACg |  |  |  |  |  |  |
|  | U | F: TTTgTgTTTTgATgTTTgTAggTTTTTgT |  | 53 |  |  | 93 |  |
|  |  | R: AACTCCACTCTTCCAAAAACAAAACA | |  |  |  |  |  |
| MSP: Methylation-specific polymerase chain reaction; M: Methylation; U: Unmethylation. | | | | |  |  |  |  |

**References**

1. Parsons DW, Jones S, Zhang X, Lin JC, Leary RJ, Angenendt P, Mankoo P, Carter H, Siu IM, Gallia GL, Olivi A, McLendon R, Rasheed BA, Keir S, Nikolskaya T, Nikolsky Y, Busam DA, Tekleab H, Diaz LA, Jr., Hartigan J, Smith DR, Strausberg RL, Marie SK, Shinjo SM, Yan H, Riggins GJ, Bigner DD, Karchin R, Papadopoulos N, Parmigiani G, Vogelstein B, Velculescu VE, Kinzler KW (2008) An integrated genomic analysis of human glioblastoma multiforme. Science 321: 1807-1812 doi:10.1126/science.1164382

2. Hegi ME, Diserens AC, Gorlia T, Hamou MF, de Tribolet N, Weller M, Kros JM, Hainfellner JA, Mason W, Mariani L, Bromberg JE, Hau P, Mirimanoff RO, Cairncross JG, Janzer RC, Stupp R (2005) MGMT gene silencing and benefit from temozolomide in glioblastoma. N Engl J Med 352: 997-1003 doi:10.1056/NEJMoa043331
